# Neglected Urban Villages in Current Vector Surveillance System: Evidences in Guangzhou, China

**DOI:** 10.3390/ijerph17010002

**Published:** 2019-12-18

**Authors:** Sijia Wu, Hongyan Ren, Wenhui Chen, Tiegang Li

**Affiliations:** 1 State Key Laboratory of Resources and Environmental Information System, Institute of Geographic Sciences and Natural Resources Research, Chinese Academy of Sciences, 11A Datun Road, Chaoyang District, Beijing 100101, China; sjwu_9407@163.com; 2 College of Geographical Science, Fujian Normal University, No.8 Shangsan Road, Fuzhou 350007, China; mymail111@163.com; 3 Department of Infectious Diseases, Guangzhou Center for Disease Control and Prevention, Guangzhou 510440, China

**Keywords:** urban villages, *Ae. albopictus* density, Neglected macroscopic incubator, vector surveillance system, Guangzhou

## Abstract

Numerous urban villages (UVs) with substandard living conditions that cause people to live there with vulnerability to health impacts, including vector-borne diseases such as dengue fever (DF), are major environmental and public health concerns in highly urbanized regions, especially in developing countries. It is necessary to explore the relationship between UVs and vector for effectively dealing with these problems. In this study, land-use types, including UVs, normal construction land (NCL), unused land (UL), vegetation, and water, were retrieved from the high-resolution remotely sensed imagery in the central area of Guangzhou in 2017. The vector density from May to October in 2017, including *Aedes. albopictus (Ae. albopictus)*’s Breteau index (BI), standard space index (SSI), and adult density index (ADI) were obtained from the vector surveillance system implemented by the Guangzhou Center for Disease Control and Prevention (CDC). Furthermore, the spatial and temporal patterns of vector monitoring sites and vector density were analyzed on a fine scale, and then the Geodetector tool was further employed to explore the relationships between vector density and land-use types. The monitoring sites were mainly located in NCL (55.70%–56.44%) and UV (13.14%–13.92%). Among the total monitoring sites of BI (79), SSI (312), and ADI (326), the random sites accounted for about 88.61%, 97.12%, and 98.47%, respectively. The density of *Ae. albopictus* was temporally related to rainfall and temperature and was obviously differentiated among different land-use types. Meanwhile, the grids with higher density, which were mostly concentrated in the Pearl River fork zone that collects a large number of UVs, showed that the density of *Ae. albopictus* was spatially associated with the UVs. Next, the results of the Geodetector illustrated that UVs posed great impact on the density of *Ae. albopictus* across the central region of Guangzhou. We suggest that the number of monitoring sites in the UVs should be appropriately increased to strengthen the current vector surveillance system in Guangzhou. This study will provide targeted guidance for local authorities, making more effective control and prevention measures on the DF epidemics.

## 1. Introduction

Dengue fever (DF), as a mosquito-borne disease caused by one of the four dengue virus serotypes (DENV 1–4), mainly transmitted to humans by mosquito vectors, including *Ae. albopictus* and *Aedes aegypti* [1,2], is prevalent in the tropical and sub-tropical regions of the world and poses a serious threat to global public health [3,4]. Approximately 94% of indigenous cases in mainland China were reported from Guangdong Province, and 83% of these cases were in Guangzhou City, where *Ae. albopictus* is the sole vector of DF transmission [5,6]. Since there were no effective vaccines or specific therapies [7,8], the key practical way to prevent the spread of the dengue virus is to eliminate the *Ae. albopictus* by preventing them from breeding, which requires an effective vector surveillance system [9,10].

In order to effectively monitor the mosquito breeding sites and feeding activities, a considerable amount of investigations on global or local mosquitoes and their influencing factors have been widely conducted in the world [11,12,13]. It has been pointed out that warm and humid conditions were suitable for mosquito breeding [14]. *Ae. albopictus* originated in the forests and bred in natural habitats [11]. However, this species has gradually adapted well to urban environments [15]. Many places, especially in cities, such as waste recycling stations with poor sanitation, water storage containers, aquatic plant pots inside rooms, are more prone to breeding these vectors [16,17,18]. 

Urbanization is a global trend that results from economic development [15]. Urban space has undergone dramatic transformation and reconstruction during the rapid urbanization of China, a large number of rural villages in the urban fringe were gradually surrounded or semi-enclosed by ever-expanding cities and become so-called urban villages (UVs) with substandard living conditions [19,20,21,22]. These informal urban settlements are widely distributed in not only the Guangzhou core areas but also other Chinese cities, for example, Shenzhen (another southern city), Wuhan (a central municipal region), Xi’an (a northwestern city), and so on [23,24]. UVs, unique areas in China, are covered to some degree by local basic public services established across these urban areas, so they are obviously different from the slums (another typical informal urban settlement) in other countries (e.g., slums in India, Mumbai, etc.) [22]. In comparison to other urban settlements or land-use types, such as the normal construction land (NCL), UVs are commonly featured by poor sanitation, lack of infrastructure [25,26,27], and serious environmental pollution due to the lack of overall planning and scientific management [23]. These characteristics of UVs may provide an ideal living environment for the breeding of *Ae. albopictus* in Guangzhou City. However, the relationship between UVs and this mosquito vector has remained little understood.

Furthermore, most of the available research about the effects on vectors has focused on the analysis at a relatively large spatial scale, such as a community, township, county, and even prefecture-level [28,29,30,31]. However, the flight distance of *Ae. albopictus* is limited [11], and an appropriate fine scale is often the final node to effectively prevent and control vector-borne diseases [23]. 

Therefore, the objectives of this study were to explore the potential influences of various land-use types on the density of *Ae. albopictus* derived from the current vector surveillance system in the central area of Guangzhou. The aim was to provide targeted guidance for local authorities effectively improving the current vector surveillance system and making control and prevention measures on the DF epidemics in urban regions with numerous UVs.

## 2. Materials and Methods 

### 2.1. Study Area

**Figure 1:**
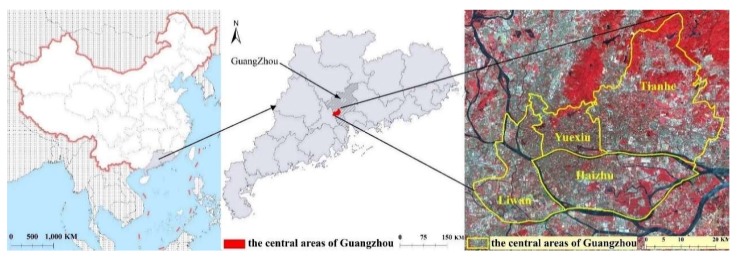
Study areas and GF-2 satellite data (Red: band5, Green: band4, Blue: band3) coverage of the four districts of Liwan, YueXiu, Haizhu, and Tianhe in Guangzhou City. (The “red” pixels mean the areas covered by vegetation. The GF-2 is the abbreviation of Gaofen-2, which is the name of a satellite image data.)

The study area was located in the central areas of Guangzhou (113°23–113°36′E, 23°08′–23°14′N) and included the four districts of Liwan, YueXiu, Haizhu, and Tianhe (Figure 1). This central area of the study with a total area of 279.63 km^2^ has a population of 5.24 million permanent residents and is also the economic center of Guangzhou [32]. In 2017, the gross domestic product (GDP) of the central area reached US $151.73 billion [33]. The characteristics of its sub-tropical monsoon climate are obvious—warm and rainy, adequate solar, and hot resources, an annual average temperature of 21–23 °C, and an average annual rainfall of 1800 mm. The city has experienced rapid expansion during the recent regional economic development [15]. These suitable natural and socio-economic conditions in Guangzhou are conducive to the breeding and activities of *Ae. albopictus*.

### 2.2. Data Collection

Generally, the density of *Ae. albopictus* is higher from May to October [34].The available vector indices data from May to October in 2017 for this study, including *Ae. albopictus’*s Breteau index (BI), standard space index (SSI), and adult density index (ADI), were obtained from the vector surveillance system implemented by Guangzhou Center for Disease Control and Prevention (CDC). Among them, the mosquito larval indices (BI and SSI) were used to estimate the density of immature *Ae. Albopictus* (larvae/pupae), while ADI was used to monitor the adult *Ae. albopictus*. Containers with immature *Ae.* mosquitoes were considered as positive containers [35].

Three vector indices used were as follows:

BI: In each residential monitoring site, any indoors and outdoors water-holding containers infested with larvae or pupae near any of not less than 100 houses in the vicinity (outside the houses, a radius of 5 m was considered as part of the property) were inspected.

(1) $$\mathrm{BI}=\frac{\mathrm{No}.\;\mathrm{of}\;\mathrm{positive}\;\mathrm{containers}\;}{\mathrm{No}.\;\mathrm{of}\;\mathrm{houses}\;\mathrm{surveyed}\;}\times 100$$

SSI: In each non-residential monitoring site (e.g., schools, factories, ports, public markets, parks, commercial zones), any indoors and outdoors water-holding containers infested with larvae or pupae were inspected. The monitors used the visual method to convert the inspection area to one standard room per 15 m^2^ and then calculated the SSI.

(2) $$\mathrm{SSI}=\frac{\mathrm{No}.\;\mathrm{of}\;\mathrm{positive}\;\mathrm{containers}\;}{\mathrm{No}.\;\mathrm{of}\;\mathrm{standard}\;\mathrm{rooms}\;\mathrm{surveyed}\;}\times 100$$

ADI: Adult *Ae. albopictus* was captured with hand-nets by monitors for 15 minutes in the four directions of each monitoring site. The number of adult *Ae. albopictus* captured, the inspected hours and the monitors were recorded accordingly.

(3) $$\mathrm{ADI}=\frac{\mathrm{No}.\;\mathrm{of}\;\mathrm{the}\;\mathrm{adult}\;Ae.\;albopictus\;\mathrm{captured}\;}{\mathrm{No}.\;\mathrm{of}\;\mathrm{the}\;\mathrm{inspected}\;\mathrm{hours}\times \mathrm{No}.\;\mathrm{of}\;\mathrm{the}\;\mathrm{monitors}}$$

BI that was only used to monitor the density of immature *Ae. Albopictus* in residential areas was supplemented with SSI, which can be employed in the no-residential areas. Meanwhile, ADI was used to estimate the population density of adult *Ae. albopictus* across the external environment of the study area [36]. In general, these three vector indices can be used to represent the density of *Ae. Albopictus* (larvae/pupae/adult mosquito) across the study area.

Referring to previous studies [37] and based on the risk level of DF transmitted by *Ae. albopictus* developed by Guangzhou CDC, *Ae. albopictus* density was divided into four levels, from low to high (Table S1). Among them, level I to IV represented low density, medium-low density, medium-high density, and high density, respectively. The address information of monitoring sites was used in conjunction with geocoding and coordinate deviation correction to obtain monitoring site data for a spatial point layer using ArcGIS Software (Version 10.5, ESRI, Redlands, CA, USA).

**Figure 2:**
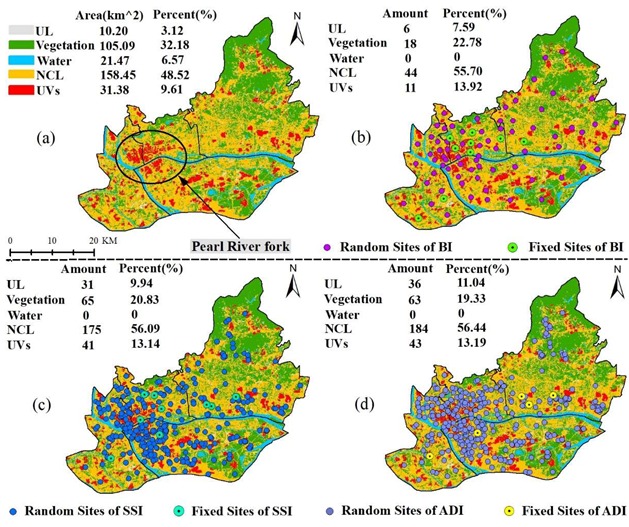
Spatial distribution of land-use types (**a**) and the monitoring sites ((**b**): the monitoring sites of BI, (**c**): the monitoring sites of SSI, and (**d**): the monitoring sites of ADI) in 2017(Amount indicates the number of monitoring sites in various land-use types. Percent indicates the proportion of monitoring sites of different land-use types in the total sites.).

Due to the habitats and activity hobbies, the spatial distribution and density of *Ae. albopictus* differed greatly among various land-use types [38]. With consideration of the high degree of urbanization of the study area, the land-use types were divided into five categories: UVs, normal construction land (NCL), unused land (UL), vegetation, and water (Figure 2a). According to the area ratio of five land-use types (Figure 2), about 58% of this region was covered by construction land (CL), including NCL (158.45 km^2^, 48.52%) and UVs (31.38 km^2^, 9.61%). In comparison, about 39% of this area was covered by permeable surfaces, including vegetation (105.09 km^2^,32.18%) and water (21.47 km^2^, 6.57%), and the remaining area was covered by UL (10.20 km^2^, 3.12%). More detailed information regarding the retrieval of the land-use types and extraction accuracy, which can meet the requirements for further analysis, can be found in an earlier study [39]. 

In addition, the abundance of vectors species generally show seasonal variation [40]. meteorological parameters such as temperature and rainfall, affect habitat productivity and *Ae. albopictus* survival, which in turn affect *Ae. albopictus* density [15,41]. Based on the daily rainfall and temperature monitoring data from May to October, the monthly average of rainfall and temperature values were calculated. The rainfall and temperature data were obtained from the Resources and Environment Science Data Center (RESDC). 

### 2.3. Spatial and Statistical Analysis

The choice of spatial scale is the basis of spatial analysis [23]. Given that the flight distance of *Ae. albopictus* ranges from 300 to 500 m [11], and the acreage of the largest UV is about 0.87 km^2^ [23], a spatial gridded unit of 1 km × 1 km is chosen as the spatial unit in this study. The gridded number of monitoring sites, gridded *Ae. albopictus* density and gridded land-use types area were calculated as the total number of monitoring sites per grid, the average of vector surveillance values per grid, and the sum of land-use types area per grid divided by the area of the grid, respectively. Then, the relationship between the UVs and vector density was analyzed on this grid scale.

The coefficient of variation was used to explore the differences of the monitoring sites for three vector indices (BI, SSI and ADI) in various land-use types. The BI, SSI, and ADI values on the gridded scale from May to October were analyzed with an independent *t*-test for proving whether each group of data was statistically significant. Meanwhile, taking the monitoring sites distributed in UVs as the main body, which were paired with the other monitoring sites (located in other land-use types) closed to them one by one. A paired *t*-test method was used to compare the average BI, SSI, and ADI values on each type and detect differences at the significance level of 0.10. In addition, Spearman correlation analysis was applied to explore the correlation between dynamic changes of *Ae. albopictus* density in UVs and in the entire study area. All of the above statistical analyses were completed in the SPSS software (Version 24.0, SPSS Inc.: Chicago, IL, USA).

### 2.4. Geodetector Model

In view of the spatiotemporal heterogeneity of the vector monitoring sites, the *Ae. albopictus* density may be affected by its potential influencing factors in different ways and to various degrees. It is appropriate to analyze the effects of land-use types on the *Ae. albopictus* density and test their significance by using a Geodetector model on the grid-scale [42,43]. The Geodetector is a relatively new statistical method that is mainly based on spatial variance analysis theory. It has been widely applied to explain the extent of factor *X*’s (various geographical or spatial phenomena) effect on the spatial differentiation of factor *Y*, such as the mosquito density [42,44]. The formula of the Geodetector was expressed as below:

(4) $$q=1-\frac{1}{N\sigma^{2}}\sum_{h=1}^{L}N_{h}\sigma_{h}^{2}$$

The study area is stratified into *h* subareas, denoted by *h* = 1, 2…, *L*, according to spatial heterogeneity (which is defined as an attribute whose statistical properties of a suspected determinant) [42]. *N* and $\sigma^{2}$ denote the number of units and the variance of the factor *Y* in the study area, respectively; $N_{h}$ and $\sigma_{h}^{2}$ denote the number of units and the variance of the factor *Y* in the subareas, respectively. The range of *q* value is from zero to one. If the determinant completely controls factor *Y*, the *q* value is one. If the determinant is completely unrelated to the factor *Y*, the *q* value is zero. Thus, the *q* value reflects the degree to which a determinant explains the prevalence of the factor *Y* [44]. When *q* value is large, the influence of factor *X* will be great.

The Geodetector model contains four modules: the risk detector, the factor detector, the ecological detector, and the interaction detector. In this study, the factor detector is used to quantify the extent of each land-use type’s effect on the observed vector indices (BI, SSI, and ADI) using *q* value, and the interaction detector is used to identify whether an interaction exists among different land-use factors or whether they can independently influence the spatial heterogeneity of the vector indices.

## 3. Results

### 3.1. Current Surveillance Systems of Ae. albopictus

During the study period, some obvious features are shown in the vector surveillance system (Table 1). The differences existed in the number of monitoring sites for different indices. The number of monitoring sites for BI, SSI, and ADI was 79, 312, and 326, respectively. In other words, the number of monitoring sites of SSI (ADI) was about four times that of BI. Accordingly, vector indices of SSI and ADI were the focus of the current surveillance system.

Furthermore, the number of monitoring sites for three indices were varied from May to October. The number of BI monitoring sites decreased initially, followed by an increase, but then decreased again, reaching a maximum in September (79) and a minimum in October (22). However, the number of monitoring sites for SSI and ADI showed the same trend in volatility, both reaching a minimum in July (66, 64), and reaching a maximum in August (124) and June (114), respectively. In addition, the unchanged location of the monitoring sites during the study period was defined as fixed sites, while the changed location of the monitoring sites was defined as random sites. As shown in Table 2, the number and proportion of random sites for BI, SSI, and ADI were 70 (88.61%), 303 (97.12%), and 321 (98.47%), respectively. These results showed that the current surveillance system was dynamic and with better mobility. 

It was found that the distribution of monitoring sites for BI, SSI, and ADI were spatially different across this region, but they were mainly concentrated in the Pearl River fork zone across Yuexiu, Liwan, and Haizhu (Figure S1). The central region of Guangzhou City typically featured impervious surfaces (i.e., NCL and UVs) according to their dominant area percentage (58.12%), especially many UVs spatially clustered in the Pearl River fork zone (Figure 2a). The proportion of monitoring sites in various land-use types was different. In comparison, NCL (55.70%–56.44%) > Vegetation (19.33%–22.78%) > UV (13.14%–13.92%) > UL (7.59%–11.04%) > Water (0). 

Meanwhile, these monitoring sites showed obvious differences in land-use types (Table 3). The values of coefficient of variation of the monitoring sites in various land-use types were 1.082 (BI), 1.076 (SSI) and 1.077 (ADI), respectively, and in various land-use types except water were 0.741 (BI), 0.735 (SSI) and 0.736 (ADI), respectively.

**Figure 3:**
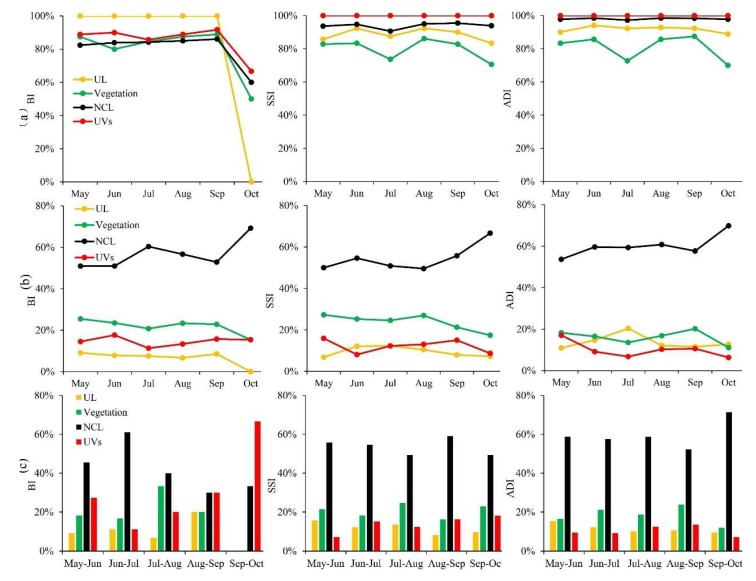
The dynamic changes in the proportion of random sites among different land-use types (**a**) The proportion of random sites in each land-use type; (**b**) The proportion of random sites in the whole region; (**c**) New random sites for each land-use type account for the sum of new random sites in the whole region.

From different perspectives to observe the dynamic changes of random sites during the study period, the number of random sites was found that featured obviously in various land-use types. Compared with the fixed sites, the proportion of random sites located in UVs was larger than other land-use types, especially for SSI and ADI (Figure 3a). However, among the total random monitoring sites of BI (SSI and ADI) in each month, UVs possessed few random sites, only accounting for about 11%–18%, (8%–16%, 6%–17%), respectively. NCL possessed many more random sites than other land-use types, accounting for more than half of the total random sites in the whole region (Figure 3b). And from May to October, new random sites added monthly were mainly distributed in the NCL regions. These sites were almost equal to the sum of new random sites added monthly in the UL, vegetation, and UVs areas (Figure 3c). That was, the current surveillance system focused on the NCL regions, but the UVs as unique areas with high population density had been ignored.

### 3.2. Temporal and Spatial Changes of Ae. albopictus Density

**Figure 4:**
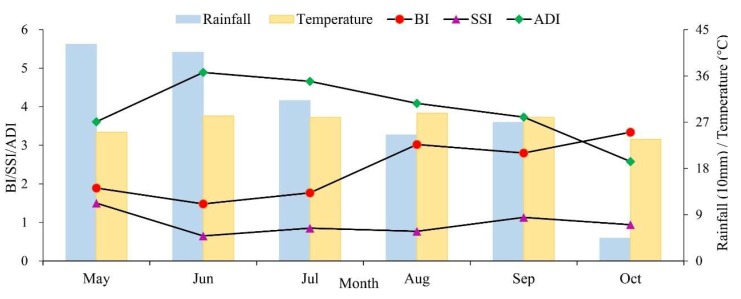
The temporal relationship between three vector indices (BI, SSI, and ADI) and meteorological factors, including rainfall and temperature.

The temporal variations of rainfall, temperature, and the *Ae. albopictus* density across the central area during the six-month-monitoring periods were shown in Figure 4. The rainfall decreased in volatility, and the temperature increased first to a maximum of 28.7 °C and finally decreased to a minimum of 23.6 °C. Under this climatic condition, BI increased in volatility, while SSI and ADI decreased in volatility. From May to July, BI and SSI both decreased first and then increased, but ADI increased first and then decreased. From July to October, BI and SSI fluctuated, while ADI continued to decrease. In a word, three vector indices (BI, SSI, and ADI) values reached their maximum in October, May, and June, respectively. These results illustrated that the monthly variations of immature and adult *Ae. albopictus* density was related to climate changes.

**Figure 5:**
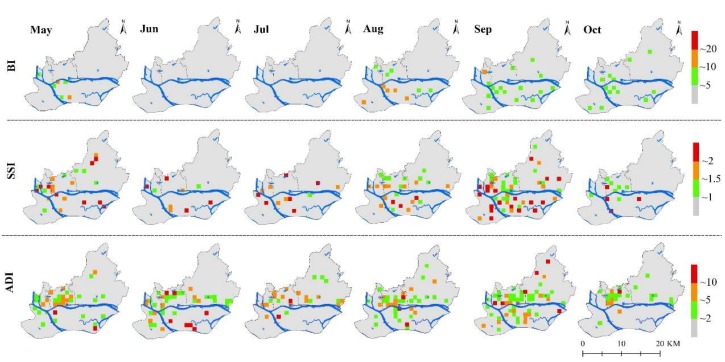
The spatial and temporal distribution of vector density (BI, SSI, and ADI) from May to October.

The results of the independent *t*-test showed that the mean value of BI, SSI, and ADI on the grid-scale were statistically significant (*p* < 0.05). It illustrated that this grid-scale was suitable for subsequent analysis. Vector indices values, especially their high values, were temporally and spatially featured on the 1 km × 1 km scale (close to the largest UV area) (Table S2, Figure 5). The number of high-value grids of SSI and ADI, and their proportions fluctuated monthly, and they reached maximums in September (18, 21.43%) and Jun (7, 11.86%), respectively, while the high-value grids of BI did not appear during the study period. As shown in Figure 5, the high-value grids of vector indices (SSI and ADI) were mainly concentrated in the Pearl River fork zone. These results showed that *Ae. albopictus* density on the grid-scale was temporally and spatially differentiated.

### 3.3. Differences of Vector Density in Various land-use Types

**Figure 6:**
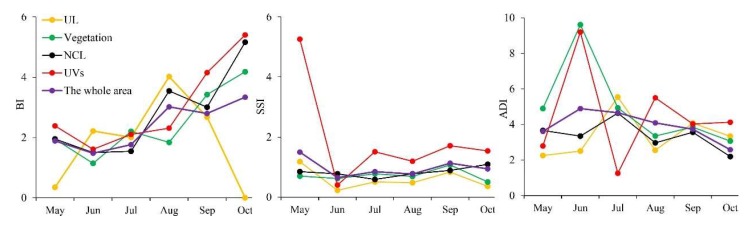
Monthly variation in three vector indices (BI, SSI, and ADI) in various land-use types.

Similar to the monitoring sites, the values of vector indices were also varied among the different land-use types (Table S3). The number of SSI and ADI monitoring sites in UVs were fewer, only 13.14% and 13.19% of the total monitoring sites, but the proportion of monitoring sites with high value (level IV) in UVs was larger than other land-use types, and respectively 1.48 and 2.53 times of the entire region. Furthermore, the dynamic changes of vector indices values in the various land-use types were also different. Compared to the UV regions, the vector indices (SSI and ADI) were relatively stable, and the vector indices (BI, SSI, and ADI) values were slightly lower in the NCL. The changes of vector monitoring values in UVs were almost consistent with those in the entire area from May to October, and the high values of the corresponding vector index appeared the same month approximately (Figure 6). 

**Figure 7:**
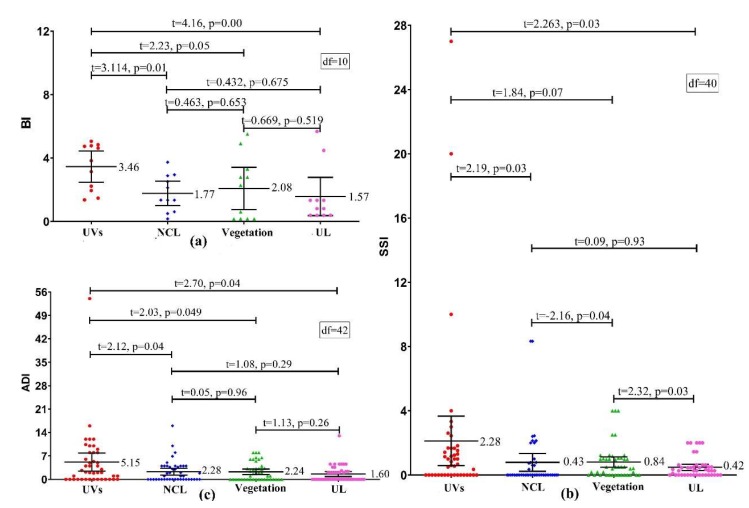
The comparison of the mean values of the vector indices in different land-use types ((**a**) BI, (**b**) SSI, and (**c**) ADI).

As illustrated in Figure 7, the orders of three vector indices values (BI, SSI, ADI) among various land-use types were as follows: UVs (3.46) > vegetation (2.08) > NCL (1.77) > UL (1.57), UVs (2.28) > vegetation (0.84) > NCL (0.43) > UL (0.42) and UVs (5.15) > NCL (2.28) > vegetation (2.24) > UL (1.60). The analysis results of the paired *t*-test had proved that the monthly average value of BI (3.46), SSI (2.28), and ADI (5.15) were significantly higher in the UVs than that in other land-use types (*p* < 0.10), respectively. In other words, the density of *Ae. albopictus* in UVs was significantly higher than that in other land-use types.

**Figure 8:**
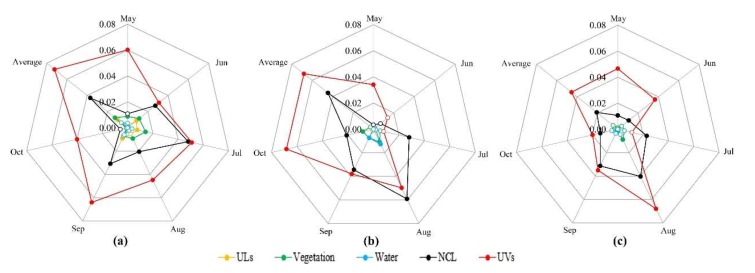
The *q* values of the factor detector from May to October ((**a**) BI, (**b**) SSI, and (**c**) ADI). The solid point means that the value is significant at the level of 0.10.

Based on the results above, the *q* values obtained from the factor detector found that different land-use types posed obviously different effects on the spatial disparities of vector indices (Figure 8). UVs could predominantly explain spatial differentiation of vector indices, followed by NCL and vegetation, whereas water and UL had minimal effects. Meanwhile, as shown in Table S4, there were interactions between the main land-use factors (UVs, NCL, and vegetation), and the explanatory power of any two factors after the interaction was manifested as a nonlinear or bilinear enhancement. Among them, the interaction relationships between UVs and NCL were a mainly nonlinear enhancement. In comparison, the interactive values of UVs and NCL were greater than the interactive values of UVs and vegetation, NCL, and vegetation. Taken together, these results demonstrated that the *Ae. albopictus* density would be affected more greatly under any two land-use factors, especially the interaction of UVs and NCL. Accordingly, the UVs played a leading role and the impacts of UVs on *Ae. albopictus* density was not only existed in the UV areas but also in NCL and vegetation regions.

## 4. Discussion

Numerous UVs and increasingly mosquito-borne disease outbreaks are major environmental and public health concerns in highly urbanized regions. In this study, the influences of five land-use types on the density of *Ae. albopictus*, were investigated on a fine-scale in the central region of Guangzhou for the first time. Several notable findings could provide useful clues for local authorities improving current vector surveillance systems aiming to prevent and control mosquito-borne diseases more effectively.

An obvious temporal variation was displayed in the density of *Ae. albopictus* across the central region of Guangzhou. Previous studies had focused on the relationship between *Ae. albopictus* density and meteorological parameters, and found that *Ae. albopictus* density was affected by regional climatic conditions [45,46]. This study was similar to previous studies: the mean values of BI and SSI in May were higher than those in June, which might be related to the sudden rainfall from the beginning of June to mid-June in Guangzhou. The outdoor water-holding containers were washed away, which led to mosquito larval indices (BI and SSI) values decreased, and the reduction of SSI values obtained many more from outdoor containers was greater than that of BI. Due to the monitoring method of ADI was less affected by the sudden rainfall, the temperature that increased from May to June caused a rise in ADI values. From June to August, the rainfall was reduced, but the environment was also humid, and the average temperature in the central region of Guangzhou was suitable for the *Ae. albopictus* breeding and activity [34]. Therefore, compared to June, the mosquito larval indices (BI and SSI) values increased in this period. In October, the rainfall dropped sharply, and the temperature gradually decreased, many mosquitoes gathered at indoor areas of warm residential regions [47,48], the BI values increased which were mainly obtained from indoor water-holding containers in residential regions, while SSI and ADI values decreased. 

However, according to previous studies, time lags might have effects on the relationships between vector densities and meteorological variables [1,49]. Additionally, another previous study had stated that the impact of rainfall on *Ae. albopictus* density may be affected by many other environmental factors [50]. If the more detailed data for vector densities can be retrieved, the more accurate relationship between vector indices and meteorological factors can be analyzed. Nevertheless, it is impossible for the current vector surveillance system to use a large amount of manpower and material resources to obtain detailed monitoring data in the actual work. Therefore, based on the existing data, the associations between vector indices and meteorological variables (temperature and rainfall) can be used to describe the changes in BI, SSI, and ADI in the study period. In general, we recommend that local authorities should adjust the vector surveillance measures according to climatic conditions in time.

In this study, the proportion of vector monitoring sites with high value in UVs were relatively more than other land-use types. It can be clearly seen that UVs possessed higher values of *Ae. albopictus* density and gridded high values were mainly located around the Pearl River fork with widely distributed UVs, developed public transportation, and a denser population [23]. This was consistent with previous findings that the vector density in urban regions might be related to disordered urban expansion, public transportation, and population density [51,52]. Furthermore, the factor detector revealed that the UVs could predominantly explain the spatial variability of *Ae. albopictus* density across the study region and interaction detector indicated that the impacts of UVs on vector density were not only existed in UVs but also in NCL and vegetation regions. There are some possible reasons for this. First, UVs, as a type of informal urban settlement, having poor sanitation, water storage containers, and aquatic plant pots inside rooms, provide *Ae. albopictus* with a suitable environment for survival and breeding [16,17,18]. Second, UVs are the best residence for *Ae. albopictus*, featuring slightly lower land surface temperature than NCL areas, which was suitable for *Ae. albopictus* breeding and having a large floating population and a high density of low-cost accommodation [39]. Third, the maximum flight distance of *Ae. albopictus* is about 500m [11]. It was not difficult to see that UVs were an important factor influencing the *Ae. albopictus* density in the central area of Guangzhou.

On the one hand, we found that the current vector surveillance system was more dynamic, but it paid more attention to NCL regions and relatively ignored the UVs which scattered in the whole region. However, the latter tended to possess higher *Ae. albopictus* density. In addition, the few monitoring sites located in UVs were almost all random sites, and there were no fixed sites, which would be detrimental to long-term monitoring of the areas where gathered many mosquitos. The results of this study indicated that the current vector surveillance system needed to be improved, and more monitoring sites, especially fixed sites, should be added in UV regions. It was similar to the topic of previous studies that focused on some targeted intervention measures (including effective vector monitoring system) that should be designed and established for preventing and controlling these vector-borne infections, especially in the regions featured by a complex environment [2,53]. On the other hand, previous studies found that adult female *Ae. albopictus* were directly involved in dengue transmission [54], but larval monitoring indices (BI, SSI) were more suitable for establishing predictive warning thresholds in the study about the prevention and control of DF epidemics [55,56,57]. While the current vector surveillance system included BI and SSI, and their monitoring sites were more random, the attention to BI was significantly lower than that of SSI. Accordingly, we proposed to enhance the capability of BI acquired in UVs and their surrounding zones.

There were some limitations to the study. First, the longer time series of more detailed *Ae. albopictus* density data should be acquired to compare and analyze the link between influential factors and BI, SSI, and ADI. And the spatiotemporally matched UVs data should be longer to verify the relationship between UVs and *Ae. albopictus* density of this study. Second, combining the longer-term sequence of the *Ae. albopictus* density and UVs information, and adding other potentially influencing factors such as human population density, a suitable prediction model should be established to estimate the distribution of *Ae. albopictus* in the UVs region. Finally, DF epidemic information should be integrated for more in-depth analysis in further research

## 5. Conclusions

As a neglected macroscopic incubator, UVs were more suitable for breeding the *Ae. albopictus* due to the lack of overall planning and scientific management. Our findings are sufficiently reasonable and detailed to illustrate that UVs posed great impacts on the density of *Ae. albopictus* across the central region of Guangzhou city. We suggest that the number of monitoring sites in the UVs should be appropriately increased to strengthen the current vector surveillance system in Guangzhou. This study will supply valuable clues to local authorities, making more effective control and prevention measures on the DF epidemics.

## Figures and Tables

**Table 1:** The number of monitoring sites for different indices from May to October.

Month	No. of Monitoring Sites of BI	No. of Monitoring Sites of SSI	No. of Monitoring Sites of ADI
May	64	97	87
Jun	59	108	114
Jul	62	66	64
Aug	69	124	112
Sep	79	122	109
Oct	22	78	68

**Table 2:** The number of random sites and fixed sites of each vector index and their proportion (%).

Indices	No. of Random Sites	The Proportion of Random Sites	No. of Fixed Sites	The Proportion of Fixed Sites
BI	70	88.61%	9	11.39%
SSI	303	97.12%	9	2.88%
ADI	321	98.47%	5	1.53%

**Table 3:** The statistical parameters of the amount of monitoring sites for three vector indices (BI, SSI, and ADI) in various land-use types.

Area	Indices	Minimum	Maximum	Mean	Standard Deviation	Coefficient of Variation
The total area	BI	0	44	15.80	17.09	1.082
	SSI	0	175	62.40	67.13	1.076
	ADI	0	184	65.20	70.21	1.077
The total area except water	BI	6	44	19.75	14.64	0.741
	SSI	31	175	78.00	57.35	0.735
	ADI	36	184	81.50	60.00	0.736

## Supplementary Materials

The following are available online at https://www.mdpi.com/1660-4601/17/1/2/s1, Figure S1. Spatial distribution of the vector monitoring sites from May to October, Table S1. Different levels of vector density monitoring indices, Table S2. The number of high-value (level IV) grids of vector indices (SSI and ADI) and their proportions (%) of non-zero grids, Table S3. The number of vector monitoring sites with high value (level IV) and their proportions (%) in various land-use types, Table S4. The dominant interactions between any two land-use factors from May to October.

## References

[1] Jing L.H., Hans S., Annelies W.S., Joacim R. (2014). Vectorial Capacity of Aedes aegypti: Effects of Temperature and Implications for Global Dengue Epidemic Potential. PLoS ONE.

[2] Telle O., Vaguet A., Yadav N.K., Lefebvre B., Daudé E., Paul R.E., Cebeillac A., Nagpal B.N. (2016). The Spread of Dengue in an Endemic Urban Milieu-The Case of Delhi, India. PLoS ONE.

[3] Mariam O.D.B., Simard F., Caprara A. (2018). Supporting and strengthening research on urban health interventions for the prevention and control of vector-borne and other infectious diseases of poverty: Scoping reviews and research gap analysis. Infect. Dis. Poverty.

[4] Bhatt S., Gething P.W., Brady O.J., Messina J.P., Farlow A.W., Moyes C.L., Drake J.M., Brownstein J.S., Hoen A.G., Sankoh O. (2013). The global distribution and burden of dengue. Nature.

[5] Lai S.J., Huang Z.J., Zhou H., Anders K.L., Perkins T.A., Yin W.W., Li Y., Mu D., Chen Q.L., Zhang Z. (2015). The changing epidemiology of dengue in China, 1990-2014: A descriptive analysis of 25 years of nationwide surveillance data. BMC Med..

[6] Gao J.R., Liu J.H., Liu J.H., Liu S.Z. (2014). Research and control strategies for Aedes albopictus in Guangzhou, China, during recent five years. Chin. J. Vector Biol. Control.

[7] Stéphanie D., Kate Z., Valéry R. (2018). Interventions for vector-borne diseases focused on housing and hygiene in urban areas: A scoping review. Infect. Dis. Poverty.

[8] Huang J.H., Su C.L., Yang C.F., Liao T.L. (2012). Molecular characterization and phylogenetic analysis of dengue viruses imported into Taiwan during 2008–2010. Am. J. Trop. Med. Hyg..

[9] Tandina F., Doumbo O.K., Yaro A.S., Traoré S.F., Parola P., Robert V. (2018). Mosquitoes (Diptera: Culicidae) and mosquito-borne diseases in Mali, West Africa. Parasites Vectors.

[10] Zhang Y.T., Wang T., Liu K.K., Xia Y., Yi L., Jing Q.L., Yang Z.C., Hu W.B., Lu J.H. (2016). Developing a Time Series Predictive Model for Dengue in Zhongshan, China Based on Weather and Guangzhou Dengue Surveillance Data. PLoS Negl. Trop. Dis..

[11] Yukiko H. (2011). Dengue Vectors and their Spatial Distribution. Trop. Med. Health.

[12] Medley K.A. (2010). Niche shifts during the global invasion of the Asian tiger mosquito, Aedes albopictus Skuse (Culicidae), revealed by reciprocal distribution models. Glob. Ecol. Biogeogr..

[13] Åström C., Rocklöv J., Hales S., Béguin A., Louis V., Sauerborn R. (2012). Potential Distribution of Dengue Fever Under Scenarios of Climate Change and Economic Development. Ecohealth.

[14] Proestos Y., Christophides G.K., Ergüler K., Tanarhte M., Waldock J., Lelieveld J. (2015). Present and future projections of habitat suitability of the Asian tiger mosquito, a vector of viral pathogens, from global climate simulation. Philos. Trans. R. Soc. Lond..

[15] Li Y., Kamara F., Zhou G., Puthiyakunnon S., Li C., Liu Y., Zhou Y., Yao L., Yan G., Chen X.G. (2014). Urbanization increases Aedes albopictus larval habitats and accelerates mosquito development and survivorship. PLoS Negl. Trop. Dis..

[16] Pang S.T., Lei X.G., Chen B.Z., Wang X., Xue W., Wu P.B. (2017). Analysis of Aedes albopictus larvae surveillance and its influence factors in Xi’an City. Chin. J. Hyg. Insectic. Equip..

[17] Yan Z.Q., Hu Z.G., Jiang Y.M. (2007). The larva distribution characteristics of Aedes albopictus population in Guangzhou. Chin. J. Vector Biol. Control.

[18] Zahouli J.B.Z., Koudou B.G., Müller P., Malone D., Tano Y., Utzinger J. (2017). Urbanization is a main driver for the larval ecology of Aedes mosquitoes in arbovirus-endemic settings in south-eastern Côte d’Ivoire. PLoS Negl. Trop. Dis..

[19] Chung H. (2010). Building an image of Villages-in-the-City: A clarification of China’s distinct urban spaces. Int. J. Urban Reg. Res..

[20] Hao P., Hooimeijer P., Sliuzas R., Geertman S. (2013). What Drives the Spatial Development of Urban Villages in China?. Urban Stud..

[21] Taubenböck H., Kraff N.J. (2014). The physical face of slums: A structural comparison of slums in Mumbai, India, based on remotely sensed data. J. Hous. Built Environ..

[22] Huang X., Liu H., Zhang L.P. (2015). Spatiotemporal Detection and Analysis of Urban Villages in Mega City Regions of China Using High-Resolution Remotely Sensed Imagery. IEEE Trans. Geosci. Remote Sens..

[23] Ren H.Y., Wu W., Li T.G., Yang Z.C. (2019). Urban villages as transfer stations for dengue fever epidemic: A case study in the Guangzhou, China. PLoS Negl. Trop. Dis..

[24] Ren H.Y., Zheng L., Li Q.X., Wu Y., Lu L. (2017). Exploring Determinants of Spatial Variations in the Dengue Fever Epidemic Using Geographically Weighted Regression Model: A Case Study in the Joint Guangzhou-Foshan Area, China, 2014. Int. J. Environ. Res. Public Health.

[25] Lin X.B., Ma X.G., Li G.C. (2014). Formation and Governance of Informality in Urban Village Under the Rapid Urbanization Process. Econ. Geogr..

[26] Li Z.G., Wu F.L. (2013). Residential Satisfaction in China’s Informal Settlements: A Case Study of Beijing, Shanghai, and Guangzhou. Urban Geogr..

[27] Wekesa B.W., Steyn G.S., Otieno F.A.O. (2011). A review of physical and socio-economic characteristics and intervention approaches of informal settlements. Habitat Int..

[28] Cunze S., Koch L.K., Kochmann J., Klimpel S. (2016). Aedes albopictus and Aedes japonicus- two invasive mosquito species with different temperature niches in Europe. Parasites Vectors.

[29] Serpa L.L.N., Gisela R.A.M.M., Lima A.P.D., Voltolini J.C., Arduino M.D.B., Barbosa G.L., Andrade V.B., Lima V.L.C. (2013). Study of the distribution and abundance of the eggs of Aedes aegypti and Aedes albopictus according to the habitat and meteorological variables, municipality of São Sebastião, São Paulo State, Brazil. Parasites Vectors.

[30] Tsai C.H., Chen T.H., Lin C., Shu P.Y., Su C.L., Teng H.J. (2017). The impact of temperature and Wolbachia infection on vector competence of potential dengue vectors Aedes aegypti and Aedes albopictus in the transmission of dengue virus serotype 1 in southern Taiwan. Parasites Vectors.

[31] Duan J.H., Li R.B., Lin W.B., Cai S.W., Lu W.C., Li J.Q., Lin H.B., Yi J.R., Liu W.H. (2008). Study on the effect of urbanization on the breeding characteristics of Aedes albopictus in residential area. Chin. J. Vector Biol. Control.

[32] Sang S.W., Chen B., Wu H.X., Yang Z.C., Di B., Wang L., Tao X.Y., Liu X.B., Liu Q.Y. (2015). Dengue is still an imported disease in China: A case study in Guangzhou. Infect. Genet. Evol..

[33] Guangzhou Economic and Social Development Statistics Bulletin 2017 Statistics Bureau of Guangzhou Municipality. http://www.gdstats.gov.cn/tjzl/tjgb/.

[34] Liang L.L., Li X.N., Luo L., Xia Y. (2019). Analysis on the density of Aedes albopictus and the risk of dengue fever transmission in Guangzhou City from 2016 to 2017. Chin. J. Hyg. Insectic. Equip..

[35] Lin C.H., Wen T.H. (2011). Using Geographically Weighted Regression (GWR) to Explore Spatial Varying Relationships of Immature Mosquitoes and Human Densities with the Incidence of Dengue. Int. J. Environ. Res. Public Health.

[36] Liang L.L., Zhang J.Y., Li X.N., Luo L. (2019). A comparative analysis of Aedes albopictus surveillance between Guangzhou emergency mosquito vector control team and district CDCs in 2016–2017. Chin. J. Vector Biol. Control.

[37] Yue Y.J., Lu L., Liu Q.Y. (2016). Relationship between mosquito density and land cover types in Guangzhou, China. Chin. J. Vector Biol. Control.

[38] Yan J., He L.H. (2017). Advances in research on impacts of geographical landscape factors on mosquito density. Chin. J. Vector Biol. Control.

[39] Wu W., Ren H.Y., Yu M., Wang Z. (2018). Distinct Influences of Urban Villages on Urban Heat Islands: A Case Study in the Pearl River Delta, China. Int. J. Environ. Res. Public Health.

[40] Bowman L.R., Runge-Ranzinger S., Mccall P.J. (2014). Assessing the relationship between vector indices and dengue transmission: A systematic review of the evidence. PLoS Negl. Trop. Dis..

[41] Roiz D., Eritja R., Molina R., Melero-Alcibar R., Lucientes J. (2008). Initial distribution assessment of Aedes albopictus (Diptera: Culicidae) in the Barcelona, Spain, area. J. Med Entomol..

[42] Wang J.F., Li X.H., George C., Liao Y.L., Zhang T., Gu X., Zheng X.Y. (2010). Geographical Detectors-Based Health Risk Assessment and its Application in the Neural Tube Defects Study of the Heshun Region, China. Int. J. Geogr. Inf. Sci..

[43] Wang J.F., Xu C.D. (2017). Geodetector: Principle and prospective. Acta Geogr. Sin..

[44] Xin X., Yuan Z., Zhang X.L., Xia S.Y. (2018). Identifying the Impacts of Social, Economic, and Environmental Factors on Population Aging in the Yangtze River Delta Using the Geographical Detector Technique. Sustainability.

[45] Pan J.Y., Ling Y.L., Zhou Y., Cai Z.L., Liu Q.L. (2017). Correlation between dengue fever epidemic and community spatial factors in Haizhu District of Guangzhou. J. Med. Pest Control.

[46] Brady O.J., Golding N., Pigott D.M., Kraemer M.U.G., Messina J.P., Jr R.C.R., Scott T.W., Smith D.L., Gething P.W., Hay S.I. (2014). Global temperature constraints on Aedes aegypti and Ae. albopictus persistence and competence for dengue virus transmission. Parasites Vectors.

[47] Zhang B. (2014). Study on the Characteristics of Mosquito Breeding in Urbanization and Its Relationship with Landscapes and Water Quality (Taking Shanghai for Example).

[48] Yan Z.Q., Hu Z.G., Jiang Y.M., Wu H.Y., Pan Z.M. (2006). Study on the application of standard space index in the population dynamic surveillance of Aedes albopictus. Chin. J. Hyg. Insectic. Equip..

[49] Cheong Y.L., Burkart K., Leitão P.J., Lakes T. (2013). Assessing weather effects on dengue disease in Malaysia. Int. J. Environ. Res. Public Health.

[50] Jiang Y.M., Yan Z.Q., Hu Z.G., Li C.L., Xu J.M., Liang X.Y. (2014). Predicting the population density of Aedes albopictus according to meteorological data. J. Trop. Med..

[51] Reiner R.C., Perkins T.A., Barker C.M., Niu T., Chaves L.F., Ellis A.M., George D.B., Menach A.L., Pulliam J.R.C., Bisanzio D. (2013). A systematic review of mathematical models of mosquito-borne pathogen transmission: 1970–2010. J. R. Soc. Interface.

[52] Hassan A.N., Nogoumy N.E., Kassem H.A. (2013). Characterization of landscape features associated with mosquito breeding in urban Cairo using remote sensing. Egypt. J. Remote Sens. Space Sci..

[53] Vikram K., Nagpal B.N., Pande V., Srivastava A., Saxena R., Anvikar A., Das A., Singh H., Tuli N.R., Gupta S.K. (2016). An epidemiological study of Dengue in Delhi, India. Acta Trop..

[54] Chang F.S., Tseng Y.T., Hsu P.S., Chen C.-D., Lian I.B., Chao D.Y. (2015). Re-assess Vector Indices Threshold as an Early Warning Tool for Predicting Dengue Epidemic in a Dengue Non-endemic Country. PLoS Negl. Trop. Dis..

[55] Thammapalo S., Chongsuvivatwong V., Geater A., Dueravee M. (2008). Environmental factors and incidence of dengue fever and dengue haemorrhagic fever in an urban area, Southern Thailand. Epidemiol. Infect..

[56] Chen S.C., Liao C.M., Chio C.P., Chou H.H., You S.H., Cheng Y.H. (2010). Lagged temperature effect with mosquito transmission potential explains dengue variability in southern Taiwan: Insights from a statistical analysis. Sci. Total Environ..

[57] Pham H.V., Doan H.T., Phan T.T., Minh N.N.T. (2011). Ecological factors associated with dengue fever in a central highlands Province, Vietnam. BMC Infect. Dis..

