# Supplementary material for: Neglected Urban Villages in Current Vector Surveillance System: Evidences in Guangzhou, China
Source: Int J Environ Res Public Health. 2019 Dec 18;17(1):2. doi: 10.3390/ijerph17010002 (PMC6981632; doi:10.3390/ijerph17010002)
Supplement: Supplementary file 1 [file ijerph-17-00002-s001.pdf]

Supplementary material:

For different vector monitoring indices (BI, SSI and ADI), the thresholds for each density level were different. Referring to previous studies and based on the risk level of DF transmitted by *Ae. albopictus* developed by CDC, *Ae. albopictus* density was divided into four levels from low to high. Among them, level I to Ⅳ were represented low density, medium–low density, medium–high density and high density, respectively.

Table S1. Different levels of vector density monitoring indices.

Indices	I	II	Ⅲ	Ⅳ
BI	≤5	5–10	10–20	>20
SSI	≤1	1–1.5	1.5–2	>2
ADI	≤2	2–5	5–10	>10

The address information of vector monitoring sites was used in conjunction with geocoding (http://www.gpsspg.com/xGeocoding/) and coordinate deviation correction to obtain monitoring sites data for a spatial point layer using ArcGIS Software (Version 10.5, ESRI, Redlands, CA, USA). From May to October, the distribution of monitoring sites for BI, SSI and ADI were spatially different across this region, but they were mainly concentrated in the Pearl River fork zone across Yuexiu, Liwan, and Haizhu.

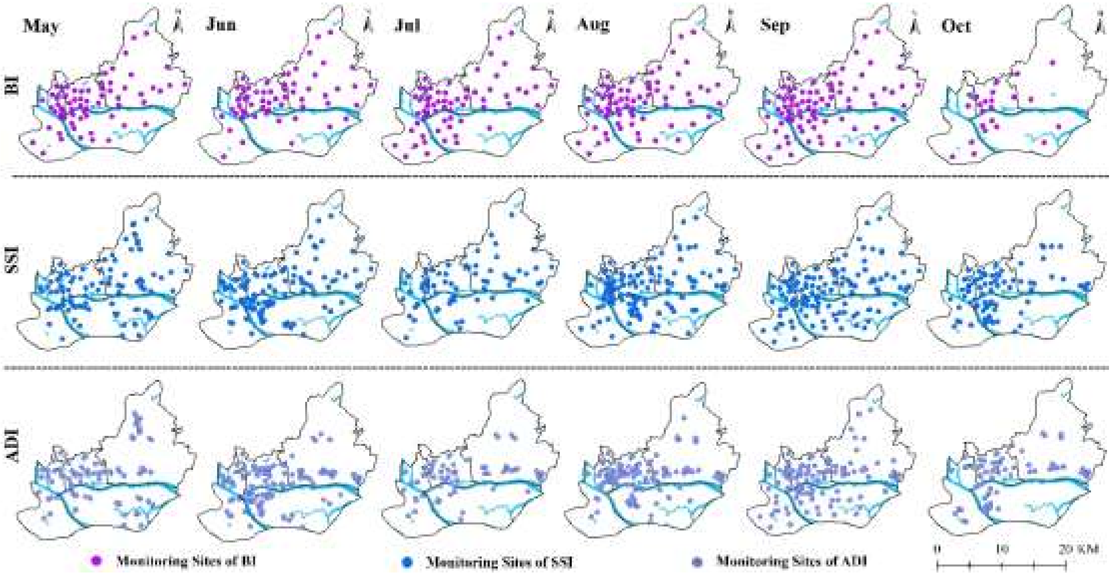

Figure S1. Spatial distribution of the vector monitoring sites from May to October.

In order to explore the dynamic variations of high–value (level Ⅳ) of vector indices on the grid scale during the study period, we counted the number of high–value grids and their percentage of non–zero grids. As shown in Table S2, there wasn't any high–value (level Ⅳ) grid of BI from May to October.

Table S2. The number of high–value (levelⅣ) grids of vector indices (SSI and ADI) and their proportions（%）of non–zero grids.

Month	SSI（Ⅳ）		ADI （Ⅳ）	
	Number	Proportion（%）	Number	Proportion（%）
May	9	15.79%	1	2.13 %
Jun	4	8.16 %	7	11.86 %
Jul	6	13.04 %	2	5.41 %

Aug	4	5.48 %	3	5.56 %
Sep	18	21.43 %	6	7.89 %
Oct	4	9.52 %	2	5.71 %

In order to analyze the differences of vector indices values among the various land–use types, we counted the number of high–value (level Ⅳ) monitoring sites and their proportions (%) in various land–use types. As shown in Table S3, there wasn't any BI monitoring site with high value (level Ⅳ) during study period.

Table S3. The number of vector monitoring sites with high value (level Ⅳ) and their proportions (%) in various land–use types.

Indices	NCLs (%)	Vegetation (%)	UVs (%)	UL (%)	Water (%)	Total (%)
SSI	24（13.71%）	8（12.31%）	8（19.51%）	1（3.23%）	0	41（13.14%）
ADI	7（3.80%）	4（6.35%）	6（13.95%）	1（2.78%）	0	18（5.52%）

According to the interaction detector, we selected the dominant interactions between any two land–use factors (UVs, NCL and vegetation) from May to October.

Table S4. The dominant interactions between any two land–use factors from May to October.

		**UVs ∩ NCL**			**UVs ∩ Vegetation**			**NCL ∩ Vegetation**		
May	BI	0.081	+	Nonlinear	0.079	+	Nonlinear	0.017	+	Bivariate
	SSI	0.049	+	Nonlinear	0.039	+	Bivariate	0.036	+	Bivariate
	ADI	0.079	+	Nonlinear	0.077	+	Nonlinear	0.021	+	Nonlinear
Jun	BI	0.068	+	Nonlinear	0.043	+	Bivariate	0.031	+	Bivariate
	SSI	0.032	+	Nonlinear	0.029	+	Nonlinear	0.012	+	Nonlinear
	ADI	0.048	+	Bivariate	0.044	+	Bivariate	0.015	+	Bivariate
Jul	BI	0.091	+	Bivariate	0.076	+	Nonlinear	0.051	+	Bivariate
	SSI	0.035	+	Bivariate	0.018	+	Bivariate	0.031	+	Bivariate
	ADI	0.042	+	Nonlinear	0.029	+	Bivariate	0.026	+	Bivariate
Aug	BI	0.080	+	Nonlinear	0.055	+	Bivariate	0.027	+	Bivariate
	SSI	0.099	+	Bivariate	0.070	+	Nonlinear	0.062	+	Bivariate
	ADI	0.079	+	Bivariate	0.075	+	Bivariate	0.043	+	Bivariate
Sep	BI	0.094	+	Bivariate	0.073	+	Bivariate	0.035	+	Bivariate
	SSI	0.083	+	Nonlinear	0.042	+	Bivariate	0.037	+	Bivariate
	ADI	0.074	+	Nonlinear	0.043	+	Bivariate	0.038	+	Bivariate
Oct	BI	0.056	+	Nonlinear	0.050	+	Nonlinear	0.013	+	Nonlinear
	SSI	0.196	+	Nonlinear	0.086	+	Nonlinear	0.026	+	Bivariate
	ADI	0.046	+	Nonlinear	0.027	+	Bivariate	0.019	+	Bivariate
monthly average	BI	0.108	+	Bivariate	0.086	+	Bivariate	0.043	+	Bivariate
	SSI	0.081	+	Bivariate	0.091	+	Bivariate	0.051	+	Bivariate
	ADI	0.068	+	Bivariate	0.051	+	Bivariate	0.027	+	Bivariate

Note:

"∩" indicates that the interactions between two factors.

"+" means that the interaction q values enhance the q values of single factor.

"+" and "Bivariate" indicates that the types of interaction is $q(X1 \cap X2) > Max(q(X1), q(X2))$

"+" and "Nonlinear" indicates that the types of interaction is $q(X1 \cap X2) > q(X1) + q(X2)$
